# Data on combination effect of PEG-coated gold nanoparticles and non-thermal plasma inhibit growth of solid tumors

**DOI:** 10.1016/j.dib.2016.08.059

**Published:** 2016-09-04

**Authors:** Nagendra Kumar Kaushik, Neha Kaushik, Ki Chun Yoo, Nizam Uddin, Ju Sung Kim, Su Jae Lee, Eun Ha Choi

**Affiliations:** a Plasma Bioscience Research Center / Department of Electrical and Biological Physics, Kwangwoon University, Seoul, 139-701 Korea; b Department of Life Science, Research Institute for Natural Sciences, Hanyang University, Seoul, 133-791 Korea

**Keywords:** Non-thermal plasma, DBD plasma, Gold nanoparticles, Cancer, Solid tumor, *in vivo* study

## Abstract

Highly resistant tumor cells are hard to treat at low doses of plasma. Therefore, researchers have gained more attention to development of enhancers for plasma therapy. Some enhancers could improve the efficacy of plasma towards selectivity of cancer cells damage. In this dataset, we report the application of low doses of PEG-coated gold nanoparticles with addition of plasma treatment. This data consists of the effect of PEG-coated GNP and cold plasma on two solid tumor cell lines T98G glioblastoma and A549 lung adenocarcinoma. Cell proliferation, frequency of cancer stem cell population studies by this co-treatment was reported. Finally, we included in this dataset the effect of co-treatment *in vivo*, using tumor xenograft nude mice models. The data supplied in this article supports the accompanying publication “Low doses of PEG-coated gold nanoparticles sensitize solid tumors to cold plasma by blocking the PI3K/AKT-driven signaling axis to suppress cellular transformation by inhibiting growth and EMT” (N. K. Kaushik, N. Kaushik, K. C. Yoo, N Uddin, J. S. Kim, S. J. Lee et al., 2016) [1].

**Specifications Table**

**Table:** 

Subject area	*Material Science, Biology, Physics*
More specific subject area	*Plasma medicine, Nano-medicine, Nanotechnology*
Type of data	*Graph, figure*
How data was acquired	*Nikon Ti-U Microscope, Biotek Synergy HT Multiwell Plate Reader,* BD FACSVerse Flow Cytometer, *ELDA Software*
Data format	*Analyzed*
Experimental factors	*PEG coated gold nanoparticles and nonthermal plasma*
	*T98G glioma and A549 adenocarcinoma cancer cell line for in vitro experiments and U87 Glioma cells for in vivo animal study*
Experimental features	*Viability, Cell morphology, Cell death by flow-cytometer, Limiting dilution assay, Tumor volume,*
Data source location	*Kwangwoon University (Plasma Bioscience Research Center) and Hanyang University (Department of Life Science), Seoul, Republic of Korea*
Data accessibility	*Data are available within this article*

**Value of the data**

• This data will be helpful for the scientific community that evaluates various nanomaterials as a cancer targeted therapy for improving existing cancer therapeutic approaches.
• This data allows the scientific community to elucidate the mechanistic basis of gold nanoparticles and plasma-induced cell destruction.
• This data confirmed that cancer stem cell populations could be suppressed through GNP and plasma co-treatment.
• This data elucidates the theses co-treatments have potential to inhibit tumor cell growth *in vivo* also.

## 1 Data

**Fig. 1:**
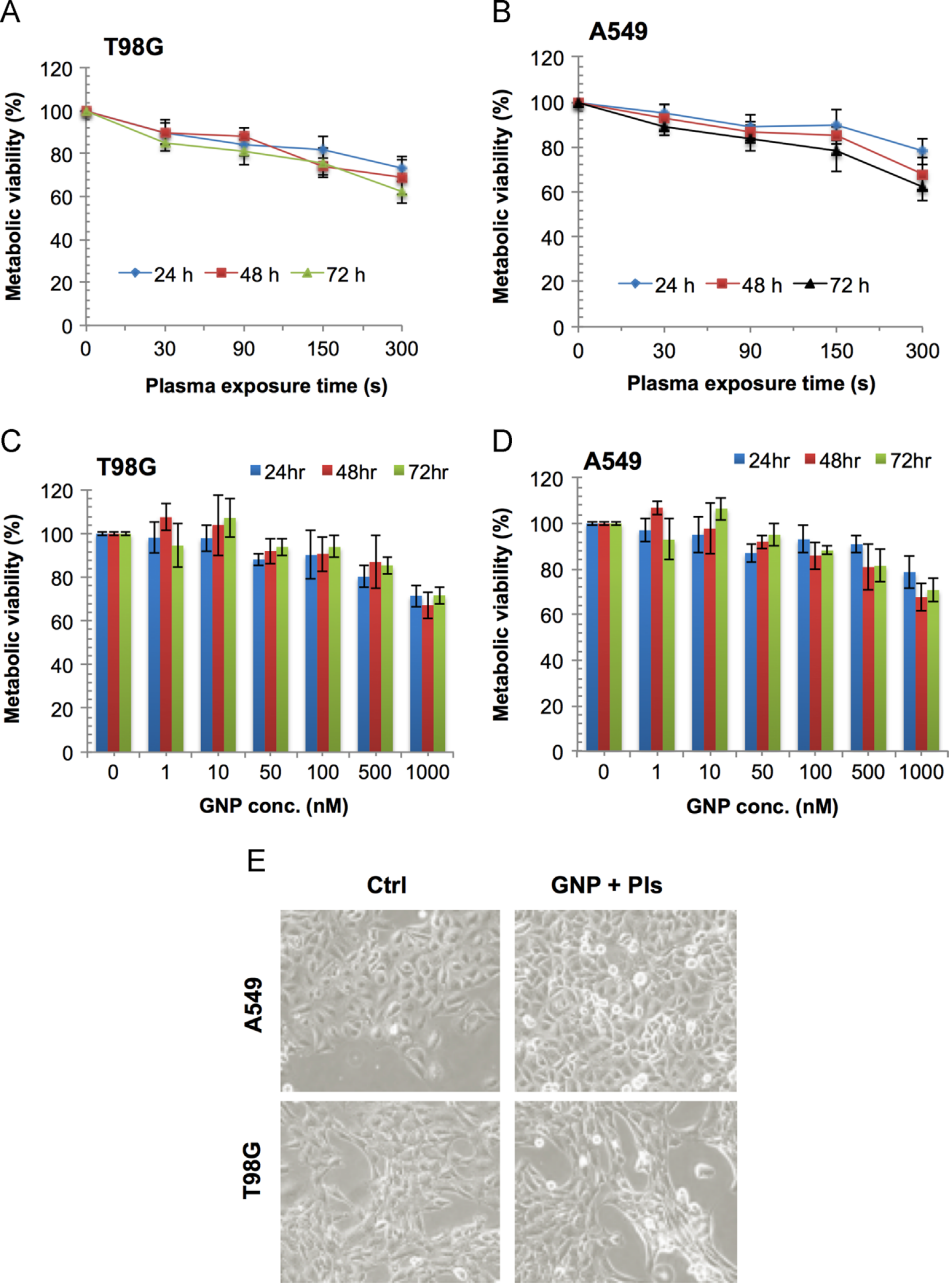
Response of gold nanoparticles and cold plasma on solid tumor cells. T98G glioblastoma and A549 lung adenocarcinoma cells which were treated with (A & B) Plasma alone in T98G and A549 cell respectively. (C & D) Gold nanoparticles (GNP) alone using different concentrations in T98G and A549 cell respectively. (E) Phase-contrast microscopic images of both cancer cell lines co-treated with GNP (100 nM) and cold plasma (150 s). Cell metabolic viability was analyzed by MTT assay following 24, 48 and 72 h using plate reader at 540 nm.

**Fig. 2:**
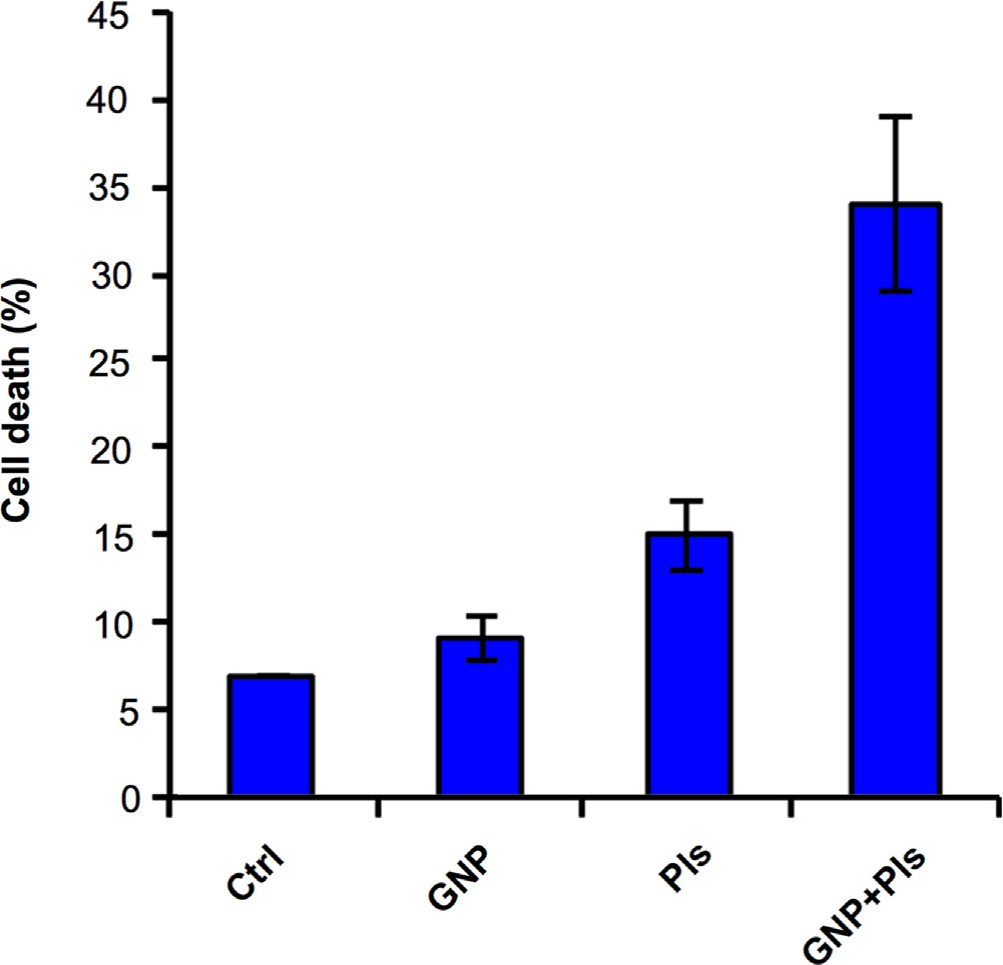
Relative cell death of 100 nM GNP and 150 s plasma (Pls) treated T98G cells as measured by flow cytometry using propidium iodide (PI) staining at similar doses following 24 h.

**Fig. 3:**
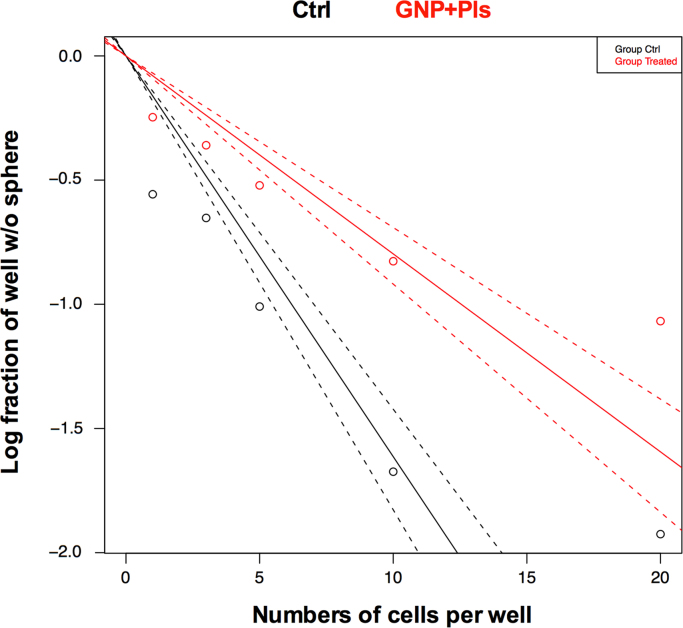
Limiting dilution assay performed on glioma cells after co-treatment with gold nanoparticles and plasma. GNP denotes gold nanoparticles and Pls denotes plasma.

**Fig. 4:**
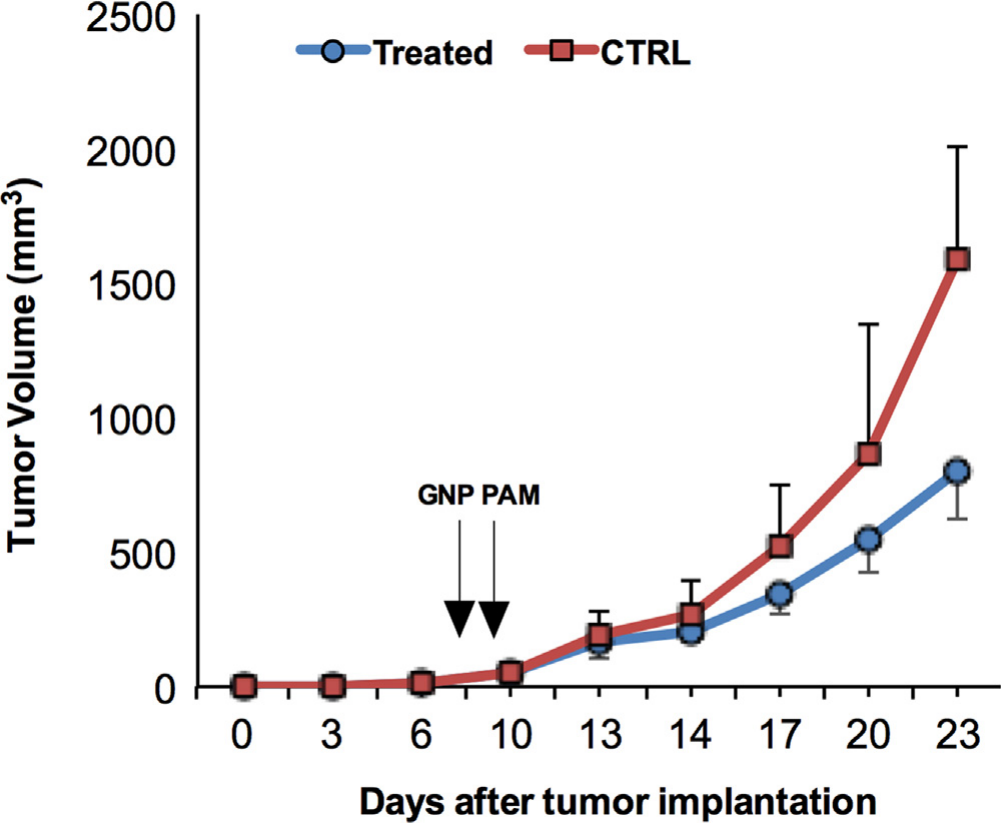
Time-dependent changes in the tumor volume in xenografted models are shown; each point on the line graph represents the mean tumor volume (mm^3^) for each group on a particular day after implantation.

We applied the combination approach to inhibit solid tumor growth using gold nanoparticles and air micro dielectric barrier discharge plasma. In this data, T98G and A549 cells decreased cell proliferation in dose and incubation time-dependent manner (Fig. 1, Fig. 2). Next, we utilized this effect on CSC maintenance, there was a decrease observed in frequency of stem cell population (Fig. 3). This dataset also depicts the analysis of tumor size *in vivo* (Fig. 4). Tumor growth was significantly inhibited in treated tumors as compared to untreated tumors.

## 2 Experimental design, materials and methods

### 2.1 Cell proliferation assay

Cell viability was determined using MTT assays in GNP plus plasma co-treated solid cancer cells. The colorimetric MTT test evaluates cell metabolic activity based on the capability of mitochondrial succinate reductase to convert a yellow-colored dye (MTT) to purple formazan in living cells. The color density of the purple-colored formazan crystals is directly proportional to the metabolic activity of the cells. MTT assays were performed according to previously reported methods [2], [3].

### 2.2 Quantification of cell death

For quantification of cell death, cells were stained with propidium iodide (PI). The cells were harvested using trypsinization, washed in phosphate-buffered saline and then incubated in PI (50 ng/mL) for 5 min at room temperature. Then immediately analyzed using BD FACSVerse using the FACS suite software.

### 2.3 Limiting dilution assay

Treated with vehicle and combination (GNP+plasma) glioma-like stem cells were enzymatically dissociated into single-cell suspensions, plated into 96-well plates with various seeding densities (0, 1, 3, 5, 10, 15, 20 cells per well). After cell seeding, the plate incubated at 37 °C for 10–15 days. On day 15, each well was observed under a microscope for the determination of individual tumor cell sphere formation. Positive wells with sphere formation were counted and log fraction of wells without spheres was plotted, and data was calculated using the Extreme Limiting Dilution Analysis (http://bioinf.wehi.edu.au/software/elda/index.html).

### 2.4 Tumor volume measurement

U87 Glioma cells (10^6^ cells) in 200 μl PBS were injected subcutaneously into right hind flan of BALB/c female nude mice. Incomplete DMEM media is treated with 10 min dielectric barrier discharge (DBD) plasma exposure to prepare plasma-activated media (PAM) [1]. Mice were treated via subcutaneous injection into tumors with 0.5 mg/kg body weight of GNP in PBS and 200 μl PAM in same group. PAM is injected four times at every next days after single dose of GNP treatment. Treated group describes treatment with both GNP and PAM. Tumor size was measured with a caliper (calculated volume=shortest diameter^2^×longest diameter/2) with 3 day intervals. This study was reviewed and approved by the Institutional Animal Care and Use Committee (IACUC) of the Center for Laboratory Animal Sciences, the Medical Research Coordinating Center, and the HYU Industry-University Cooperation Foundation.

## References

[1] Kaushik N.K., Kaushik N., Yoo K.C., Uddin N., Kim J.S., Lee S.J. (2016). Low doses of PEG-coated gold nanoparticles sensitize solid tumors to cold plasma by blocking the PI3K/AKT-driven signaling axis to suppress cellular transformation by inhibiting growth and EMT. Biomaterials.

[2] Kaushik N., Uddin N., Sim G.B., Hong Y.J., Baik K.Y., Kim C.H. (2015). Responses of solid tumor cells in DMEM to reactive oxygen species generated by non-thermal plasma and chemically induced ROS systems. Sci. Rep..

[3] Kaushik N., Lee S.J., Choi T.G., Baik K.Y., Uhm H.S., Kim C.H. (2015). Non-thermal plasma with 2-deoxy-D-glucose synergistically induces cell death by targeting glycolysis in blood cancer cells. Sci. Rep..

