# Supplementary material for: Data on combination effect of PEG-coated gold nanoparticles and non-thermal plasma inhibit growth of solid tumors
Source: Data Brief. 2016 Sep 4;9:318–23. doi: 10.1016/j.dib.2016.08.059 (PMC5026707; doi:10.1016/j.dib.2016.08.059)
Supplement: Supplementary file 1 — Supplementary material [file mmc1.docx]

Authors declare no conflict of interest

(Conflicts of interest: none)


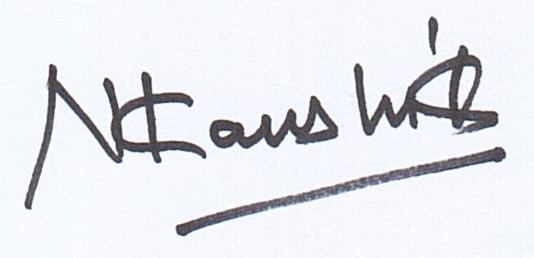


Nagendra Kaushik (PhD)

(Corresponding Author)

Assistant Professor,
Plasma Bioscience Research Center &

Department of Electrical and Biological Physics

Kwangwoon University

20 Kwangwoongil, Wolgye Dong 447-1
Seoul 01897  Korea
e-mail: [kaushik.nagendra@kw.ac.kr](mailto:kaushik.nagendra@kw.ac.kr) & [kaushik.nagendra@gmail.com](mailto:kaushik.nagendra@gmail.com)
Mobile 010-4187-8618
Office +82-2-940-8618,  Fax +82-2-940-5664
